# Multimodal Intelligent Flooring System for Advanced Smart‐Building Monitoring and Interactions

**DOI:** 10.1002/advs.202406190

**Published:** 2024-08-22

**Authors:** Yuqi Chen, Jianlong Hong, Yukun Xiao, Huiyun Zhang, Jun Wu, Qiongfeng Shi

**Affiliations:** ^1^ Joint International Research Laboratory of Information Display and Visualization School of Electronic Science and Engineering Southeast University Nanjing 210096 China

**Keywords:** flooring system, human–machine interaction, multimodal, smart building, triboelectric nanogenerator

## Abstract

The floor constitutes one of the largest areas within a building with which users interact most frequently in daily activities. Employing floor sensors is vital for smart‐building digital twins, wherein triboelectric nanogenerators demonstrate wide application potential due to their good performance and self‐powering characteristics. However, their sensing stability, reliability, and multimodality require further enhancement to meet the rapidly evolving demands. Thus, this work introduces a multimodal intelligent flooring system, implementing a 4 × 4 floor array for multimodal information detection (including position, pressure, material, user identity, and activity) and human–machine interactions. The floor unit incorporates a hybrid structure of triboelectric pressure sensors and a top‐surface material sensor, facilitating linear and enhanced sensitivity across a wide pressure range (0–800 N), alongside the material recognition capability. The floor array is implemented by an advanced output‐ratio method with minimalist output channels, which is insensitive to environmental factors such as humidity and temperature. In addition to multimodal sensing, energy harvesting is co‐designed with the pressure sensors for scavenging waste energy to power smart‐building sensor nodes. This developed flooring system enables multimodal sensing, energy harvesting, and smart‐sport interactions in smart buildings, greatly expanding the floor sensing scenarios and applications.

## Graphical abstract

The multimodal intelligent flooring system can realize the simultaneous detection of position, pressure, and material information, as well as interactive energy harvesting with a minimalist interconnection design. After the integration with neural networks, the multimodal intelligent flooring system achieves the recognition functionalities of user identity and motion, enabling the fusion of multimodal sensing and intelligent interactions in smart‐building applications.

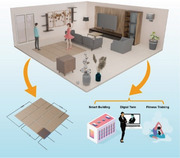

## 1 Introduction

The metaverse is a digital space for real‐time interaction, communication, and integration between the virtual and real world.^[^ ^1^, ^2^ ^]^ The concept was first proposed in the 1992 novel Snow Crash and can be realized through technological means such as augmented reality/virtual reality (AR/VR),^[^ ^3^, ^4^ ^]^ blockchain, digital twin, and human–machine interface. Among them, digital twin is a technology that maps the physical parameters of real objects, such as position, shape, gesture, and temperature, into the virtual space, thereby achieving the real‐time monitoring and controlling of the physical objects remotely. Recently, digital twin has exhibited its potential applications in broad scenarios, including online medical care,^[^ ^5^, ^6^, ^7^ ^]^ digital factory,^[^ ^8^ ^]^ and smart building.^[^ ^9^, ^10^, ^11^ ^]^ The key to realizing the digital twin requires comprehensive and effective information exchange between the real and virtual worlds. This requires a wide variety of sensors and actuators to collect physical information and interact with the real world, especially wearable sensors for monitoring the human body and environmental interaction sensors that respond to external stimuli from the surrounding environment.^[^ ^12^, ^13^, ^14^, ^15^ ^]^ Moreover, sensors must evolve to have multimodality, mutual interaction, and high security, in accordance with the characteristics of digital twins.

The smart‐building digital twin platform necessitates the deployment of a multitude of discrete sensors, which places significant demands on the energy‐supplying system and requires an easily accessible and sustainably renewable energy source. Due to the distributed nature of the sensors, the traditional centralized energy supply experiences a significant loss, which does not apply to the smart‐building scenarios. Consequently, a discrete and localized energy supply is necessary. Rechargeable batteries or supercapacitors can be employed to provide power for a certain period. However, their limited lifespan, inadequate capacity, environmental pollution, and the existence of spontaneous combustion as well as other shortcomings^[^ ^16^, ^17^, ^18^ ^]^ render them unsuitable for the energy supply needs of the digital twin platform in smart buildings. Sustainable energy sources such as solar^[^ ^19^, ^20^, ^21^ ^]^ and biofuel cells^[^ ^22^, ^23^ ^]^ are environmentally friendly, but they are dependent on external ambient conditions such as light and temperature. Triboelectric nanogenerator (TENG) is a low‐cost, universally applicable, and high‐efficiency energy supply method with promising applications in both sensing^[^ ^24^ ^]^ and energy harvesting.^[^ ^25^, ^26^ ^]^ It is also well‐suited for large‐scale array construction, enabling extensive coverage and high scalability.^[^ ^27^ ^]^ The materials available for preparing TENGs are highly diversified, ensuring efficient manufacturing and cost‐effectiveness.^[^ ^28^ ^]^ Meanwhile, the various operational modes of TENGs including contact‐separation, sliding, single‐electrode, and freestanding triboelectric layer modes, offer great design and application flexibility.^[^ ^29^ ^]^ Besides, TENG floor sensors can avoid the privacy leakage problem that may be caused by previous smart buildings relying on camera surveillance. To date, the technology has been developed for a range of applications across diverse fields, including wearable electronics,^[^ ^30^, ^31^ ^]^ smart transportation,^[^ ^32^, ^33^ ^]^ and smart buildings.

In the context of smart‐building digital twin applications, floor represents an unavoidable element of every room. As a result, it is a potential candidate for incorporation into the smart buildings sensing layer, particularly in the areas of gait pattern detection, indoor activity monitoring,^[^ ^34^ ^]^ healthcare,^[^ ^35^ ^]^ smart control,^[^ ^36^ ^]^ energy harvesting, etc. In contrast to traditional floor systems, which are limited in scale, costly to implement, consume significant amounts of power, and require complex device configurations, smart floor systems based on triboelectric sensors can readily achieve large‐scale arrangements at reduced costs due to their advanced self‐powered sensing capabilities. He et al.^[^ ^37^ ^]^ have proposed a composite square‐frame floor sensor combining planar contact and aluminum ball vibration for kinetic motion sensing such as basketball collisions and sudden falls of elderly people. Ma et al.^[^ ^38^ ^]^ developed a 3D honeycomb‐structured woven fabric TENG for pressure and external material sensing. For array realization, Yu et al.^[^ ^39^ ^]^ have proposed a pressure‐sensitive and large‐scale carpet for self‐powered fall detection. The prepared core‐shell yarn is woven into an array, with the spacing of neighboring TENGs designed according to the length of a person's foot. The electrodes are connected to each TENG separately, and localization and fall monitoring are performed based on the signals generated. Subsequently, Yang et al.^[^ ^40^ ^]^ have developed a robust TENG‐based mat array (four sets of mats), each set comprising six parallel‐connected pixels with interdigital electrode patterns in variable width ratios. The unique electrode design and the resulting ratiometric readout method enable large‐scale arrays to achieve accurate position sensing and walking trajectory monitoring.

However, recent research on smart flooring systems has been limited to unimodal sensing mechanisms, which are unable to effectively capture the full interaction information of gait, such as contact area, material, and pressure. The unimodal nature of the sensing information affects the stability, accuracy, and application range of the whole system. The full interaction information of a person's gait encompasses not only the change process of the contact area between the sole and the floor, but also the change process of their pressure. Furthermore, the array realization of smart flooring systems is important to achieve large‐scale applications in smart buildings. The traditional array design will greatly increase the complexity of the system when large arrays are used, with limitations such as large data reading latency, complex reading circuits, and difficulties in data analysis. The recent ratiometric readout method can only detect the position of a user in the array but not the pressure,^[^ ^40^ ^]^ thus requiring further improvement. In addition, the design of circuits for the separate reading of multimodal signals, which consist of position data and pressure data, requires the consideration of different circuitry.

Herein, a multimodal intelligent flooring system is developed for simultaneous realization of multimodal monitoring (including position, pressure, material, user identity and activity) and interactive energy harvesting based on a 4×4 array with a minimalist unit interconnect design. Table S1 (Supporting Information) compares the proposed flooring system to previously reported flooring systems. Ultra‐elastic triboelectric pressure sensors with unique hybrid microstructure are designed to achieve high sensitivity over a large pressure range from 0 to 800 N, exhibiting better sensitivity than previous triboelectric sensors with a comparable linear sensing range.^[^ ^41^, ^42^, ^43^, ^44^ ^]^ Furthermore, they can also be used for energy harvesting to compensate for the overall power consumption of the system and drive environment sensor nodes. The material recognition sensor is integrated on top of the pressure sensors, employing a modified interdigital electrode layout to overcome the influence of interaction modes and provide stable sensing. This integration design enables the floor unit with multimodal sensing capability for more accurate information acquisition and diversified interactions. Then an advanced array interconnection is implemented using a voltage‐division method that can simultaneously achieve the position and pressure information without the influence of ambient humidity and temperature, with minimalist output channels. The decoupling of the position and pressure information is realized by an integration algorithm based on the same input signals. In the end, user identification and smart‐sport interactions are demonstrated to show the practical applicability of the flooring system. Therefore, the proposed multimodal and intelligent flooring system can significantly broaden the sensing and interactive limits of traditional flooring systems, promoting more diverse applications in smart buildings, digital twins, smart sports, and beyond.

## 2 Multimodal Intelligent Flooring System

**Figure 1:**
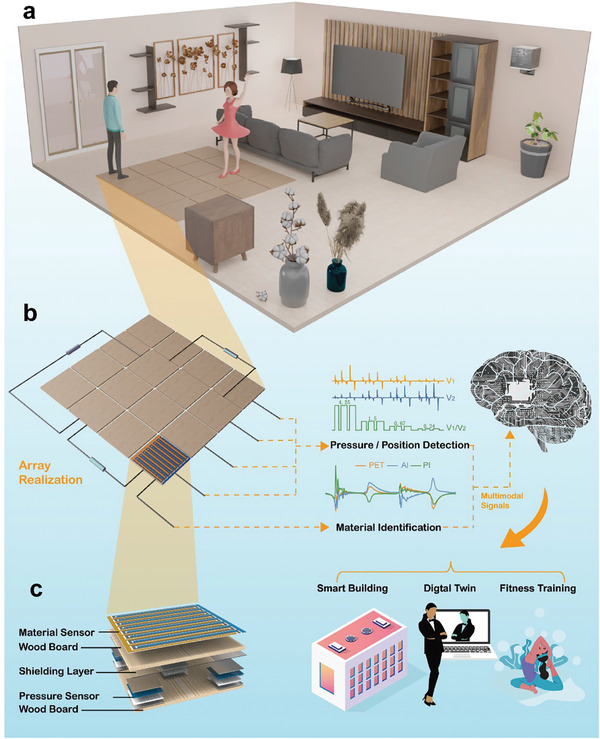
a) An overview of a smart‐building scenario enabled by the multimodal intelligent flooring system. b) The array realization and data flow of the flooring system. c) The stacking structure of a single floor unit.

As shown in **Figure** **1**a, a multimodal intelligent flooring system comprising 16 floor units is designed to demonstrate its smart‐building monitoring and interactive capabilities. To illustratively interpret its multimodality, Figure 1b shows the array realization of the floor system. When the user contacts the floor, the resulting multimodal signals containing position, pressure, and material information are generated by the sensing electrodes, which are then processed and fed into a neural network for user identity and activity recognition, thus realizing the application of smart buildings, digital twins, and fitness training. Figure 1c depicts the stacking structure of a single floor unit, which consists of a material‐recognition sensor and pressure sensors. The material‐recognition sensor at the top surface is in direct contact with the user's foot to gather information about the contact area and material. The triboelectric pressure sensors are designed for pressure sensing and energy harvesting. The shielding layer consists of an insulating polyethylene glycol terephthalate (PET) thin film and a conductive aluminum layer. The aluminum layer is grounded to isolate the output signals from the triboelectric pressure sensors and the material sensor. This prevents the crosstalk between pressure and material signals and ensures the accuracy of subsequent data processing. The pine board on top of the shielding layer is relatively lightweight, which reduces the impact on the performance of the pressure sensing.

## 3 Design and Characterization of Pressure Sensor

**Figure 2:**
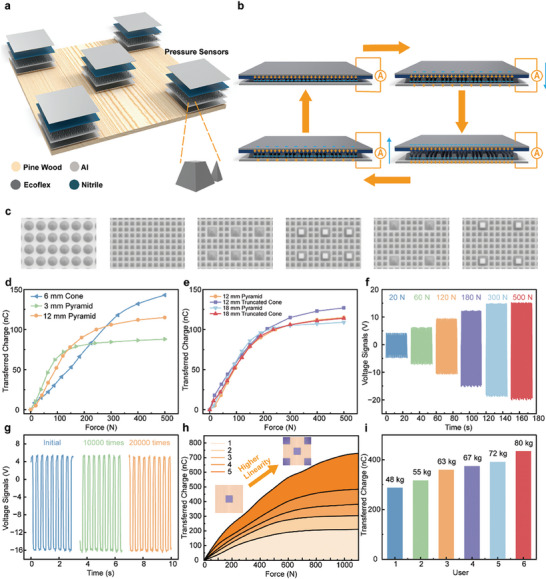
The design and characterizations of a floor unit. a) A floor unit with an enlarged view of the microstructure. b) The operation principle of the floor sensor. c) The schematic illustration of the six kinds of 3D microstructures. d, e) The transferred charges of ultra‐elastic triboelectric pressure sensors with different microstructures under varying applied forces from 0 to 500 N. f) The voltage output of the ultra‐elastic triboelectric pressure sensor with truncated cone‐pyramid hybrid structure. g) Reliability test of the triboelectric pressure sensor. h) The transferred charges of a floor unit with varying numbers of ultra‐elastic triboelectric pressure sensors. i) The measurement result of 6 users with different weights.

Pressure monitoring is a crucial aspect of smart‐building applications and serves as the primary component of multimodal sensing. The pressure signal provides information such as weight, position, and type of motion. **Figure** **2**a,b shows the schematic of the ultra‐elastic triboelectric pressure sensor and its contact‐separation mode for power generation. The sensor adopts nitrile as the positive triboelectric material, silicone rubber (Ecoflex 00–30) as the negative triboelectric material, and aluminum as the electrodes. When external pressure is applied, the positive and negative materials come into contact, causing electrons to transfer from the surface of the positive material to the surface of the negative material due to their different electron affinity. The charge density of the two materials is the same. When the external pressure is released and the two materials are separated, a potential difference is generated between the two electrodes, inducing charges flowing through the external load (transient current) to achieve electrostatic equilibrium. When the external pressure resumes, the induced charge returns to the initial electrode, generating a transient current in the opposite direction. Therefore, with cyclic applied pressure, a periodic electrical output will be produced correspondingly.

Within the pressure sensor, the negative triboelectric layer is made of Ecoflex with a 3D microstructure, which can increase the effective contact area of the triboelectric interface under pressure to achieve better output performance. A rational 3D microstructure design is required to guarantee excellent performance including sensitivity, linearity, and maximum sensing range. In order to determine the optimal structure of the negative triboelectric layer for smart building application scenarios, six different structures are investigated as shown in Figure 2c, where the overall dimensions of the triboelectric layer are 10 cm × 10 cm, the bottom interconnection layer is 1 mm thick, and the microstructure has a height of 4 mm. Full details are shown in Figure S1 (Supporting Information). The first structure is a conical single microstructure with a bottom diameter of 5 mm and a center spacing of 6 mm. The selection of a conical structure over a large pyramid is predicated on the premise that the conical structure is more stable and better defined over a broader range of linear distances. The second structure, also a single microstructure with smaller dimensions, is a small pyramidal microstructure with a bottom edge length of 2 mm and a center spacing of 3 mm. The third structure is a hybrid microstructure consisting of a small pyramidal structure with a bottom edge length of 2 mm and a large pyramidal structure with a bottom edge length of 5 mm mixed together, and the pitch of the large structure is 12 mm. Figure 2d shows the transfer charge curves of the three kinds of structures under the action of pressure from 0 to 500 N. The transfer charge is selected for characterization purposes because it can be used to reflect the entirety of the contact and separation process. The magnitude of the transfer charge is solely contingent upon the magnitude of the force applied, not influenced by the speed of the contact and separation, which offers greater stability. Upon observing the curves in Figure 2d, it is evident that the single cone structure, with a larger individual structure, exhibits good toughness and support. Additionally, it maintains a linear transfer charge curve from 0 to 300 N, with a large saturation value. However, its sensitivity performance is poor. The transfer charge curve shows that the single small pyramid structure has the highest sensitivity due to its fastest increase of contact area when squeezed. This is evident by its largest slope between 0 and 90 N, indicating the fastest accumulation of transfer charge. Similarly, the contact area saturates quickly due to its easily deformable nature, resulting in a narrower linear interval and smaller saturation charge value. Based on the transfer charge curve of the hybrid pyramid structure, it is apparent that arranging mixed structures in the contact layer is a compromise that enables the sensor to achieve the required sensing range with better sensitivity. Therefore, the hybrid structure is a superior choice. In addition, three other hybrid structures are investigated, with the fourth one replacing the large pyramid in the hybrid pyramid structure with a truncated cone structure with a bottom edge length of 5 mm and an upper/lower edge ratio of 1/2. The fifth and sixth structures reduce the density of the large structure based on the first two hybrid structures by increasing the center spacing to 18 mm. The pressure test is conducted on these three hybrid structures and the transferred charge curves of the four hybrid structures are compared, as shown in Figure 2e. From the results, it can be seen that the truncated cone hybrid structure exhibits superior toughness compared to the pyramid hybrid structure. The sensor demonstrates a wider linear range and larger saturation charge value for the same number of microstructures. Additionally, the top planar contact surface of the sensor allows for faster charge accumulation when a smaller force is applied. When comparing the transfer charge curves of two sets with the same hybrid structure but different microstructure densities, the sensor exhibits a larger linear range and saturation charge value with lower sensitivity when the microstructures have the same shape and are more numerous. Therefore, the 3D microstructure of the negative triboelectric layer is chosen as a truncated cone‐pyramid hybrid structure with a center spacing of 12 mm after a comprehensive investigation. The voltage profile of the sensor constructed using this structure is tested under different pressures, and a voltage of 35 V can be achieved under a pressure of 500 N, as shown in Figure 2f. To verify the operational stability, a repeatability test is conducted (Figure 2g). The sensor's sensing performance does not show any significant degradation after 20000 cycles. Furthermore, Figure S2 (Supporting Information) illustrates the response time, recovery time, and current signal. The response time is defined as the time required for the charge signal to reach saturation, while the recovery time is defined as the time required for the signal to return to its original state. Figure S2a (Supporting Information) illustrates that the response time and recovery time are 82 ms and 71 ms, respectively. Figure S2b (Supporting Information) demonstrates that the current signal of the pressure sensor is positively correlated with pressure.

In the scenario of the multimodal intelligent floor system, the floor unit must withstand a pressure equivalent to the weight of a normal adult. Previous tests have indicated that a single ultra‐elastic triboelectric pressure sensor normally cannot meet this requirement. Therefore, the number of pressure sensors needs to be increased. To determine the optimal number of sensors, we conducted a pressure test on the floor unit. The floor unit consisting of 1, 2, 3, 4, and 5 ultra‐elastic triboelectric pressure sensors is investigated. The transfer charge curves are shown in Figure 2h. The results indicate that as the number of sensors increases, the linearity and sensing ranges of the floor unit also increase. With only one triboelectric sensor, the transferred charge saturates at around 300 N, and the saturation charge value is small. As the number of pressure sensors increases, the linear range and saturation charge value also increase. When the number of sensors increased to five, they exhibit good linearity in the range of 0 to 800 N and do not saturate, meeting the requirements for the self‐powered intelligent flooring system. When the applied force exceeds 800 N, the transfer charge curve begins to deviate from linearity, ultimately reaching a saturation point around 1100 N. To ensure uniform stress and maintain balance, we placed the five sensors in the four corners and the center position. Figure 2i displays the floor unit activities generated by the transfer of charge for six users with varying body weights. The charge signal allows for easy differentiation between the six users.

## 4 Energy Harvesting Co‐Design

**Figure 3:**
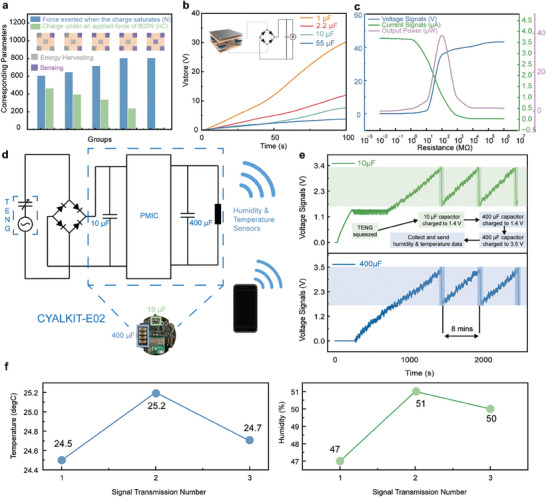
The co‐design of energy harvesting with pressure sensing. a) The transferred charges of the floor unit with varying numbers of energy harvesters. b) Energy harvesting performance for charging capacitors with different capacitance values. c) The output current (green), voltage (blue), and output power (purple) versus resistance. d) The customized circuit for driving the temperature‐humidity sensor. e, f) Wirelessly received temperature and humidity signals using the captured energy.

In a discrete smart‐building system, there are numerous energy‐consuming sensor nodes. To enable power generation for other sensors, a portion of the ultra‐elastic triboelectric pressure sensors can be used for energy harvesting, forming the energy harvesting and pressure sensing co‐design. To determine the optimal number of sensors for energy harvesting without affecting the pressure sensing capability, a pressure test is conducted on the floor unit equipped with five ultra‐elastic triboelectric pressure sensors. Some of these sensors are used for energy harvesting. The results in **Figure** **3**a illustrate the saturated pressure (blue bar) applied to the floor unit and the charge generated for energy harvesting (green bar). When four sensors are used for energy harvesting, the floor unit has the highest charge for energy supply. However, the pressure sensing reaches saturation at around 600 N. It is also noticeable that its linearity is considerably improved compared to the case when there is only one sensor in Figure 2h. This is due to the fact that although there are four sensors that are not used for pressure sensing, their physical support can still effectively slow down the deformation rate of the negative triboelectric layer, which in turn improves the linearity of the sensors. As fewer sensors are used for energy harvesting, the charge generated for energy supply decreases gradually. This results in an increase in the linear range of pressure sensing. The experimental results align with the theoretical predictions. For optimal combined performance of energy harvesting and pressure sensing, the configuration with the center pressure sensor for energy harvesting and the four‐corner pressure sensors for pressure sensing is adopted for later applications. By connecting the energy harvesting output to a rectifier circuit, the capacitor can be charged by cyclical operation of the floor unit. The voltage for capacitors of 1, 2.2, 10, and 55 µF are observed as 30.2, 11.9, 7.6, and 3.8 V, respectively, over a period of 100 s when the experimenter steps on the floor, as shown in Figure 3b. This demonstrates the potential of the floor unit to serve as a power source for smart‐building systems. To evaluate the triboelectric output power in relation to external resistance, we continuously increased the external resistance value, as shown in Figure 3c. As the external resistance value increases, the output voltage also shows an increasing trend. At the resistance value of 100 MΩ, the peak output power of approximately 39 µW is observed.

The energy harvesting output is then used to drive the operation of a temperature‐humidity sensor with Bluetooth signal transmission when the user is active on the floor array. Figure 3d depicts the customized circuit of the energy harvesting function. The electrical output generated by the ultra‐elastic triboelectric pressure sensor first supplies energy to a small 10 µF capacitor and then the power management circuit starts to supply power to the 400 µF capacitor when the voltage on the 10 µF capacitor reaches 1.4 V, as shown in Figure 3e. The voltage on the 10 µF capacitor fluctuates between high and low levels of 1.4 V. As a result, the voltage on the large capacitor and the 400 µF capacitor also fluctuates when the voltage on the 10 µF capacitor reaches 1.4 V. At this point, the 10 µF capacitor begins to supply power to the 400 µF capacitor. When the voltage on the 10 µF capacitor and the 400 µF capacitor both reach 1.4 V, they increase synchronously until they reach 3.4 V. At this point, the device sends out information about the temperature and humidity in the room. The temperature and humidity signals are transmitted approximately once every 8 min (Figure 3f), which is in line with the requirements for smart‐building applications.

## 5 Design and Characterization of Material Sensor

**Figure 4:**
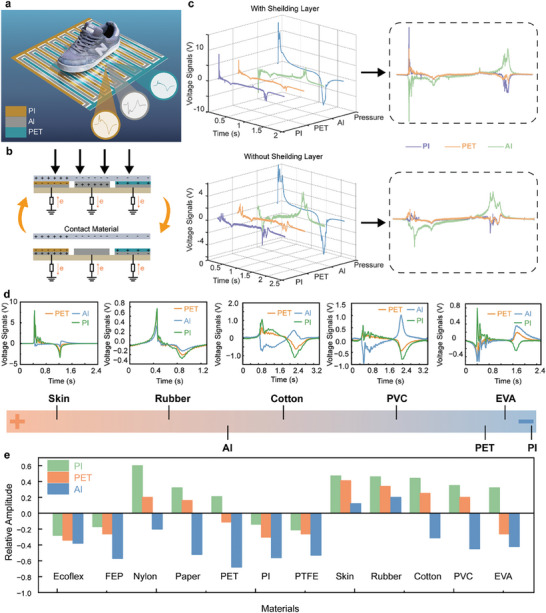
The design of the material sensor. a) Overview of material recognition function. b) The operation principle of the material sensor. c) The signals generated by the material sensor and the pressure sensor with (upper) and without (lower) a shielding layer. d) The voltage output of the material sensor in contact with different footwear materials. e) Relative amplitude of output when the material sensor contacts different materials.

In addition to pressure sensing, a planar material sensor is integrated on top of the floor unit to enable multimodal sensing. This material sensor is basically three single‐electrode‐mode triboelectric nanogenerators with different friction materials combined together, as shown in **Figure** **4**a. The material sensor is only designed at the entrance floor unit of the floor array, to maintain the balance between a succinct flooring system and the required functionality. Triboelectric signal generation requires two materials with different electron affinities. Additionally, when different materials with varying electronegativity come into contact with the same material, they produce different electrical signals. These signals can be used to determine the type of material in contact based on the output polarity and signal magnitude. The electrode array, which is covered with triboelectric layer materials of varying electronegativity, is in direct contact with the user's foot for material recognition. The material sensor is structured as interdigital electrodes, they are fixed on a 40 cm × 40 cm PET film with a middle snake electrode of the same width. These electrodes are made of aluminum and have a width of 1 cm each. In the three types of electrodes, a segment of spacing is left to prevent conduction. The two interdigital electrodes are covered with PET and Polyimide (PI) films, respectively. These films, along with the uncoated aluminum in the center, make up the three materials used to identify unknown contact materials. In Figure S3 (Supporting Information), the specific way in which the three electrodes are composed is shown. The electrode width is designed as small as 1 cm, allowing the user's foot to evenly contact all the three materials simultaneously with similar contact areas, regardless of the orientation and position of the foot. This ensures a strong output signal and greatly improves recognition accuracy. When the foot makes contact with the material sensor, a set of unique output signals is generated due to the different electron affinity of the foot and the triboelectric materials, as depicted in Figure 4b. Experiments are conducted to investigate whether crosstalk occurs between the voltage signals generated by the material sensor and the pressure sensor. The results are presented in Figure 4c under the conditions with or without a shielding layer. For the same unknown material, signals generated by contact with PI and PET are difficult to distinguish without a shielding layer (shown in Figure 4c). The observed discrepancy can be attributed to the fact that the dielectric constant of the wood board allows the conduction of displacement current. This results in the crosstalk of material signals with the pressure signal below, thereby affecting the accuracy of the results. Therefore, adding a shielding layer is necessary to prevent crosstalk between multimodal triboelectric sensors. The shielding layer serves two purposes, separating the two sensors by means of a PET film and further preventing the displacement‐current crosstalk by a grounded aluminum layer. Based on the application scenario of the multimodal intelligent flooring system, contact tests are conducted on barefoot and common sole materials, as shown in Figure 4d. The testing materials include barefoot skin, rubber, cotton, polyvinyl chloride (PVC) used in slippers, and ethylene vinyl acetate (EVA) commonly found in athletic shoes and slippers. When evaluating these materials, it is important to note that if the triboelectric material of the sensor is more electronegative than the tested material, a positive peak will be generated upon contact. For example, the three curves obtained from the experiment with rubber exhibit a positive peak followed by a negative peak, indicating that the electronegativity of rubber is less than that of the three triboelectric materials. The signal magnitude of the electrical signals generated by the material sensor in contact with the skin is much larger than that of the other materials. This distinctive feature can distinguish it from rubber. In addition, the relative amplitude of the signals can also be employed to ascertain the electronegativity of the material. The signal generated by the cotton in contact with PI is relatively stronger than that generated by contact with PET and Al. Conversely, the signal generated by the PVC in contact with Al is relatively stronger. In conclusion, a ranking of material electronegativity can be derived as follows: skin < rubber < Al < cotton < PVC < PET < EVA < PI.

To verify the working performance of the material sensor, we tested it with common triboelectric materials in the laboratory, such as Ecoflex 00–30, nylon, paper, PET, PI, and polytetrafluoroethylene (PTFE), in addition to the materials mentioned above for real‐world applications. The signals generated by the materials are normalized based on their positive‐negative relationship and magnitude, and their relative magnitudes are shown in Figure 4e. It is worth noting that the signals generated by PET and PI in contact with the same materials on the material sensors are very small, which is consistent with the theory.

## 6 Minimalist Interconnection Design

**Figure 5:**
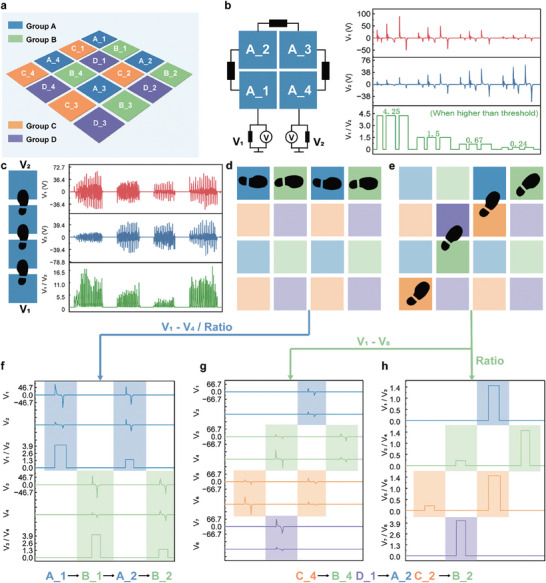
Investigation of the grouping and interconnection scheme for the floor units. a) The schematic illustration of the arrangement of the floor units into four groups. b) The connection scheme of four floor units in a group. c) The output signals when one foot steps on two floor units in the same group, showing unrecognizable output signals. d, f) The walking trajectory and the corresponding triboelectric outputs for horizontal walking. e, g, h) The walking trajectory and the corresponding triboelectric outputs for inclined walking.

Obtaining the user's position information and pressure distribution is crucial for enabling subsequent interactive functionalities. The multimodal flooring system is configured into a 4 × 4 array with 16 floor units, as illustrated in **Figure** **5**a. Traditionally, each floor unit is separated with individual output electrode, which allows for easy realization of the positioning function. However, the excessive number of electrodes increases the difficulty of designing acquisition circuits and greatly reduces the scalability of the floor array. The previous minimalist floor connection method of coding through the ratio of interdigital electrodes^[^ ^40^ ^]^ is only able to achieve positional information, but not pressure data. Therefore, it is necessary to propose a new interconnection methodology to realize simultaneous detection of both position and pressure. Dividing a portion of the floor units into a set of shared output channels is a logical method to reduce the total output channel. Within each output channel, several floor units forming a group are connected in series with one another. As shown in Figure 5b, four floor units are connected in series using serial interconnection. The two adjacent floor units are connected through a resistor of 500 KΩ, resulting in a total of three resistors with the same resistance for the whole group. The first and last units are connected to two acquisition nodes, and the input resistance of the signal acquisition circuit is also 500 KΩ. Taking the case of stepping on the floor unit A_1, the voltage measured by V_1_ on the resistor is equivalent to that of the four resistors on the right. V_2_ represents the voltage on one of the resistors, and at this point, V_1_/V_2_ is approximately 4. Similarly, when stepping on the floor unit A_2, the two left resistors have the same voltage as the three resistors on the right side, resulting in V_1_/V_2_ being approximately 1.5. In the other two cases of stepping on A_3 and A_4, V_1_/V_2_ is approximately 0.67 and 0.25, respectively. The equivalent circuits of the signal detection are shown in Figure S4 (Supporting Information). Additionally, **Table** **1** lists the theoretical values of the ratios for different positions. To verify the theoretical analysis, a pressure test is conducted on the floor group A with four connected units. Three sets of experimental data (100, 400, and 700 N) are collected with the voltage curves and voltage ratio shown in Figure 5b. To ensure the accuracy of voltage ratios, a voltage threshold of 1 V is set. Only voltage values exceeding the threshold are used to calculate V_1_/V_2_. As shown in Figure 5b, when stepping on A_1, the ratio value remains around 4.25, regardless of whether it is under the pressure of 100, 400, or 700 N. This result is consistent with the theoretical analysis. The resulting ratios for the other three positions are also consistent with the theory. The average values and standard deviations of the ratios are shown in Figure S5 (Supporting Information). The excellent distinguishment between different floor units shows the feasibility of localizing the interactive positions within the floor array by monitoring the voltage ratios between two output electrodes. This method requires only two electrodes to obtain the exact position of a set of floor units, which can be easily scaled up by connecting more floor units into one group through resistors.

**Table 1:** Theoretical value of output ratios for group positioning.

Group	Output ratio	Stepping on 1	Stepping on 2	Stepping on 3	Stepping on 4
**A**	V_1_/V_2_	4	1.5	0.67	0.25
**B**	V_3_/V_4_				
**C**	V_5_/V_6_				
**D**	V_7_/V_8_				

Meanwhile, the output signals from the two output electrodes also contain the pressure information. The interaction between the user and the floor unit leads to varying amplitude and width of the voltage signals that are relative to the speed and pressure of contact separation. This makes it difficult to intuitively analyze the difference in pressure value. On the other hand, the total transferred charge is directly determined by the pressure, which can be calculated through the integral of output current (proportional to voltage). Hence, the voltage values from the two electrodes are integrated and summed to obtain the final charge value for pressure indication, as shown in the Equation (1):

(1) $$Q\;=\frac{1}{R}\int \left(V_{1}+V_{2}\right)dt$$

where *Q* is the transfer charge, *R* is the measurement resistance, and *V_1_* and *V_2_* are the output voltages. When grouping floor units, it is important to note that two adjacent units cannot be in the same group. This is because when a person walks on the array, the front foot and back foot have opposite gait states. For instance, a walking pattern where the front foot applies pressure on the floor unit while the rear foot is lifted can result in an unrecognizable and irregular signal if both floor units belong to the same group. This happens because the positive signal generated by the pressure is canceled out by the negative signal generated by the release of pressure, as shown in Figure 5c. Based on this, the grouping shown in Figure 5a is performed to ensure that the floor units in each 2 × 2 array are not in the same group. This avoids the problem of signal crosstalk and enables the localization of arbitrary positions. Figure S6 (Supporting Information) illustrates the detailed electrode connection method and unit arrangement. To verify the grouping arrangement scheme, the user walks along the floor array in the order of the trajectories shown in Figure 5d. The output signals generated by two groups of floor units corresponding to the trajectories are shown in Figure 5f. The voltage signals exceeding the threshold value of each floor array are taken to calculate the ratio. It can be observed that the calculated ratios are all within the ideal ratio's definition range, indicating that this grouping arrangement is feasible for arbitrary walking trajectory monitoring. Although leaving and stepping down motions occur simultaneously, the signals generated in different groups do not affect each other. This arrangement also supports inclined walking. Every two neighboring diagonal units come from a different set, allowing for a more complex inclined trajectory as illustrated in Figure 5e. In this trajectory, one step covers either one or two units. The voltage signals generated by the stepping motions are observed, and the corresponding voltage ratios are computed, as shown in Figure 5g,h. These ratios are also well‐matched with the defined ratios. By considering the electrode terminals and output ratios, the floor array can be easily scaled up for monitoring arbitrary positions and walking trajectories in larger areas. As demonstrated in Figure S7 (Supporting Information), the connection schematic of a larger floor array (8×8) is shown with the same number of output electrodes, indicating its high scalability in practical applications.

## 7 Neural Network Analysis for Identity Recognition and Motion Recognition

**Figure 6:**
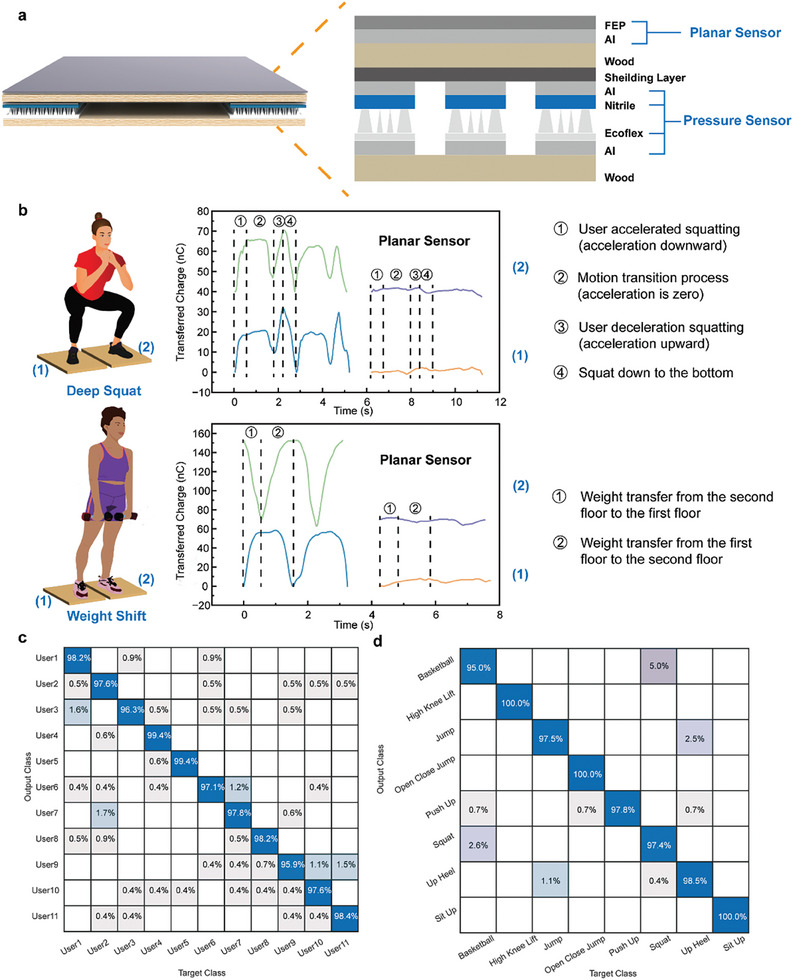
Neural network assisted intelligent recognition. a) The structure of a floor unit with surface‐mounted planar sensor and pressure sensors. b) The output signals from deep squatting and weight shifting on two floor units. c) The confusion map of the identity prediction for 11 users. d) The confusion map of the motion prediction for 8 types of motions.

After obtaining the user's pressure and position information, the multimodal intelligent flooring system analyzes the user's movement information. Certain in‐house activities do not result in an obvious change in the contact area between the foot and the floor, which poses a challenge for planar triboelectric sensors that rely on changes in the contact area to generate distinct signals.^[^ ^27^, ^40^, ^45^ ^]^ In this regard, the ultra‐elastic triboelectric pressure sensor with a 3D structure has a significant advantage in this aspect. As shown in **Figure** **6**a, a planar sensor made of FEP and aluminum is added to the surface of a floor unit with ultra‐elastic triboelectric pressure sensors for comparison. Figure 6b illustrates the output signals generated from deep squatting and weight shifting on the two floor units with planar sensors and pressure sensors. The user's feet remain stationary during deep squatting and weight shifting, resulting in a corresponding change in pressure on the floor. Therefore, the ultra‐elastic triboelectric pressure sensors generate signals that clearly distinguish the different phases of a motion. However, since the contact area of the foot with the planar sensors remains relatively constant throughout these two motions, the signals from the planar sensors are inconspicuous.This reveals the limitations of the planar sensor in such type of motion detection. The detailed voltage signals are shown in Figure S8 (Supporting Information). By combining the output signals from the pressure sensors with a neural network, the multimodal intelligent floor system can detect a wider range of motion types, increasing its practical application value.

Long short‐term memory (LSTM) is a recurrent neural network with state memory, which has a multilayer cellular structure. The use of LSTM can efficiently convey and represent information in long time series without ignoring useful information from the past. In this work, LSTM is used to assist identity recognition and motion recognition. Due to the different number of floor units involved in different types of motions, it is desirable to have a common way of inputting the acquired signals. The entire floor array has four groups of output electrodes (two in each group), and the larger signals in each group are selected and combined to form a 4‐channel input. For identity recognition, 11 users are involved to form a diverse dataset, and the confusion map is shown in Figure 6c, with a classification accuracy of 97.8%. As for motion recognition, 8 common motions, including basketball, high‐knee lift, jump, open‐close jump, push up, squat, up heel, and sit up, are categorized with 98.3% accuracy, as shown in Figure 6d.

## 8 Multimodal Intelligent Flooring System Enabled Smart‐Building Applications

**Figure 7:**
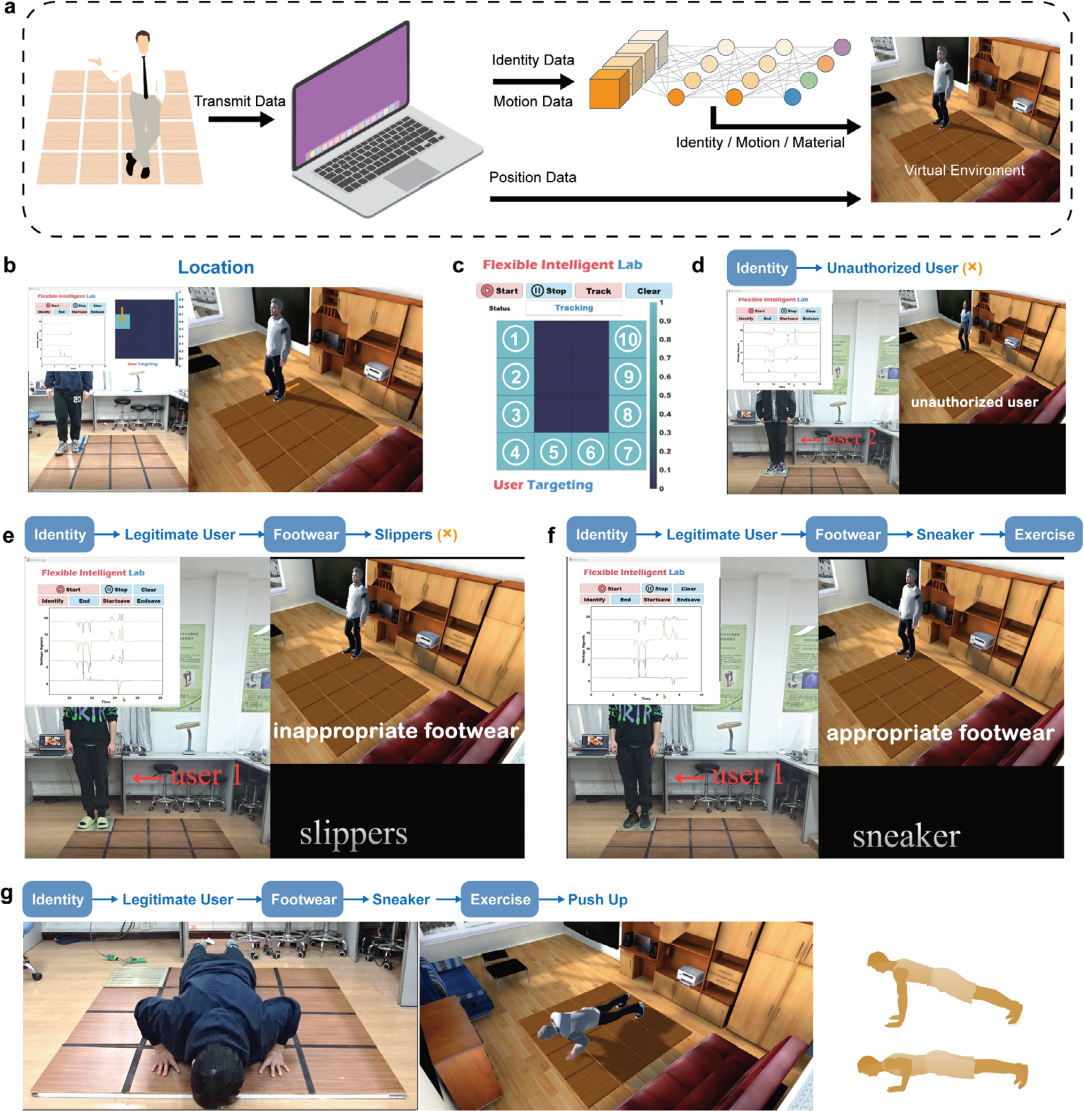
Smart‐sport interactions between the real space and virtual space in a smart‐building digital twin platform. a) The schematic illustration of the signal flow. b) The user localization by the generated signals and the movement synchronization of the avatar in the virtual space. c) The recorded user's movement trajectory. d–f) User recognition and foot material recognition as the user enters the floor array, with appropriate exercise recommendations given. g) Type of movement recognition (push‐ups as an example) and synchronization of the avatar movement in the virtual space.

To enhance the visualization of the multi‐functionality of the multimodal intelligent flooring system, a smart‐building digital twin platform is built for better demonstration of the monitoring and interactions. **Figure** **7**a illustrates the system's working scheme, where the voltage signals that are generated by the multimodal intelligent flooring system are collected by the computer. The data analysis algorithm then performs integral operation and neural network recognition to achieve user localization, identity recognition, material recognition, and motion monitoring. The intelligent flooring system relies primarily on the user's position to enable multi‐functional applications. The user localization function is demonstrated in Figure 7b and Video S1 (Supporting Information). When a user walks on the floor array, the application program interface displays the voltage signals corresponding to the interactive floor groups. The user's position in the array is displayed through a heat map, and a virtual character is synchronized and moved in the virtual space. The user's walking trajectory can be tracked by mode conversion and displayed in the program interface, as shown in Figure 7c.

In the smart‐building application, exercise advice is provided to the user based on identity and material recognition. The workflow of the application is shown in Figure 7d–f, and Video S2 (Supporting Information). When the user walks onto the entrance floor unit with the material sensor, the identity signal captured by the ultra‐elastic triboelectric pressure sensors is inputted into the neural network to determine whether it is a legitimate user. If an unauthorized user is detected, the incorrect character image is displayed in the virtual space and material recognition ceases, as shown in Figure 7d. If the user is legitimate, such as user 1 in Figure 7e, the virtual space displays the correct character and the material sensor captures the material signal for recognition. This determines whether the user's footwear is suitable for exercising. If a flip‐flop is detected, exercising is not recommended (Figure 7e). If a sneaker is detected, exercising is recommended (Figure 7f). After confirming identification and footwear, the user can exercise on the floor array for training. Figure 7g displays the virtual character performing push‐ups in synchronization with the user. This synchronization is achieved by combining information from the 4 channels into the LSTM neural network. Video S3 (Supporting Information) showcases additional exercises such as jumping and squatting.

## 9 Conclusion

In summary, a multimodal intelligent flooring system is successfully developed based on the rational integration of multi‐dimensional information capture and neural network data analytics. The entire system includes a total of 16 floor units, with each performing pressure sensing and energy harvesting simultaneously through a sensing and energy harvesting co‐design using microstructured triboelectric sensors. A unique hybrid microstructure with large truncated cones and small pyramids is adopted to achieve excellent performance in terms of sensitivity, linearity, and sensing range of 0–800 N. To realize a higher dimension of multimodal information acquisition, the floor unit located at the entrance of the floor system is configured with a material sensor. The interdigital electrode layout of the material sensor enables good signal robustness against different interactive manners such as varying stepping orientation and position. Then an advanced grouping and interconnection scheme is developed to achieve a scalable floor array with minimalist output channels as well as simultaneous trajectory monitoring and pressure sensing. The grouped interconnection design solves the limitation of not being able to cover two neighboring floor units at the same time, which is fundamental to practical applications. The algorithm to decouple the multimodal position and pressure information is obtained by time‐domain ratio calculation and summed integral analysis. Since the ambient parameters will affect the voltages at both ends, the signal analysis by ratio calculation can effectively avoid the problem that triboelectric signals are susceptible to the influence of temperature and humidity. To improve the artificial intelligence of the multimodal flooring system, a neural network is integrated for deeper signal analytics and output classification. Such integration enables the flooring system with the recognition capability of user identity and motion type, which can then be used for the real‐time synchronization of the user in the physical space and the avatar in the virtual space, facilitating the realization of a smart‐building digital twin platform. This digital twin platform includes information on the user's access privileges, dress code, location, walking trajectory, and movement status. The potential applications of this technology can be further extended to digital check‐ins, digital physical education classes, personal training, etc. The developed flooring system with the capability of multimodality detection and interactive intelligence shows broad applications in smart buildings, where it can serve as a sensing base for digital twins and incorporate artificial intelligence and metaverse technologies to create an efficient and immersive experience. Nevertheless, there is still potential for enhancement in this floor system. The materials utilized in the construction of TENG can be subjected to further investigation with the objective of attaining enhanced performance. Furthermore, nano structures can be incorporated into the existing micro structures of the triboelectric layer to enhance the signal quality. In order to reduce the energy consumption of the system, more and hybrid energy harvesting modules can be designed to improve the energy harvesting efficiency, and a sleep and walk‐up circuitry can be designed for the signal readout. Additionally, the sensing capability of the system can be extended through the incorporation of other sensing mechanisms, such as the use of pyroelectrics for temperature detection.

## 10 Experimental Section

### Fabrication of the Ultra‐Elastic Triboelectric Pressure Sensor

The key to the preparation of the ultra‐elastic triboelectric pressure sensor lies in the microstructure formation of the negative triboelectric layer, as shown in Figure S9 (Supporting Information). First, Ecoflex 00–30 solution A and solution B are mixed in the beaker according to the weight or volume ratio of 1:1, and stirred thoroughly to ensure uniform mixing. Then, the mixed solution is poured into the mold prepared by 3D printing, and the vacuum treatment is carried out to ensure that the air bubbles in the solution are all removed. Next, the solution is heated under the condition of 50 °C for 5 h. Finally, the negative triboelectric layer can be peeled off from the mold. By fixing an Al thin‐film electrode on the back of the prepared negative triboelectric layer and using the nitrile film as the positive triboelectric layer, the ultra‐elastic triboelectric electrical pressure sensor in the contact‐separation mode can be obtained.

### Fabrication of the Material Sensor

First, a number of Al foils with a width of 1 cm and a suitable length is cut. The interdigital electrodes then are extended from both sides of the PET substrate, while the snake electrode is placed in the middle. PI and PET films are attached to the interdigital electrodes.

### Characterization of the Triboelectric Output

The multimodal intelligent flooring system's output voltage is captured and logged using an oscilloscope (DSO7034B, Agilent Technologies) with a 10 MΩ impedance. The transferred charge from the ultra‐elastic triboelectric pressure sensor is measured by an electrometer (Keithley 6514), with the signals visualized and recorded via the oscilloscope. The force applied to the ultra‐elastic triboelectric pressure sensor is quantified using a force gauge (Pubtester, TST‐01H).

### Processing of Virtual Space Interaction

The voltage signals generated by the multimodal intelligent flooring system are processed in real‐time by MATLAB App Designer. The LSTM model is also developed in MATLAB. With regard to digital twin smart‐building VR interaction, the processed signals are sent to the VR scenario, which is developed based on 3D Unity, for controlling purposes.

## Conflict of Interest

The authors declare no conflict of interest.

## Supporting information

Supporting Information

Supplemental Video 1

Supplemental Video 2

Supplemental Video 3

## Data Availability

The data that support the findings of this study are available from the corresponding author upon reasonable request.

## References

[1] A. Davis , J. Murphy , D. Owens , D. Khazanchi , I. Zigurs , J. Assoc. Inf. Syst. 2009, 10, 90.

[2] K. M. de Ramos , Climate Energy 2022, 38, 22.

[3] S.‐M. Park , Y.‐G. Kim , IEEE Access 2022, 10, 4209.

[4] M. Sparkes , New Sci. 2021, 251, 18.

[5] M. N. Kamel Boulos , P. Zhang , J. Pers. Med. 2021, 11, 745. 34442389 10.3390/jpm11080745 PMC8401029

[6] M. Alazab , L. U. Khan , S. Koppu , S. P. Ramu , M. Iyapparaja , P. Boobalan , T. Baker , P. K. R. Maddikunta , T. R. Gadekallu , A. Aljuhani , IEEE Consum. Electron. Mag. 2023, 12, 29.

[7] J. N. Acosta , G. J. Falcone , P. Rajpurkar , E. J. Topol , Nat. Med. 2022, 28, 1773. 36109635 10.1038/s41591-022-01981-2

[8] Q. Min , Y. Lu , Z. Liu , C. Su , B. Wang , Int. J. Inf. Manage 2019, 49, 502.

[9] X. Zhou , K. Sun , J. Wang , J. Zhao , C. Feng , Y. Yang , W. Zhou , IEEE Trans. Industr. Inform. 2023, 19, 2684.

[10] H. Xia , Z. Liu , M. Efremochkina , X. Liu , C. Lin , Sustain. Cities Soc. 2022, 84, 104009.

[11] L. Li , B. Lei , C. Mao , J. Ind. Inf. Integr. 2022, 26, 100289.

[12] S. H. Ko , J. Rogers , Adv. Funct. Mater. 2021, 31, 2106546.

[13] F. Wen , Z. Sun , T. He , Q. Shi , M. Zhu , Z. Zhang , L. Li , T. Zhang , C. Lee , Adv. Sci. 2020, 7, 2000261. 10.1002/advs.202000261 PMC7375248 32714750

[14] T. Zhan , K. Yin , J. Xiong , Z. He , S. T. Wu , iScience 2020, 23, 101397. 32759057 10.1016/j.isci.2020.101397 PMC7404571

[15] C. Y. Yiming Liu , Z. Song , Y. Huang , K. Yao , T. Wong , J. Zhou , L. Zhao , X. Huang , S. K. Nejad , M. Wu , D. Li , J. He , X. Guo , J. Yu , X. Feng , Z. Xie , X. Yu , Sci. Adv. 2022, 8, eabl6700. 35030019 10.1126/sciadv.abl6700 PMC8759751

[16] Y. Wang , Y. Song , Y. Xia , Chem. Soc. Rev. 2016, 45, 5925. 27545205 10.1039/c5cs00580a

[17] Y. Yang , X. Liu , Z. Dai , F. Yuan , Y. Bando , D. Golberg , X. Wang , Adv. Mater. 2017, 29, 1606922. 10.1002/adma.201606922 28627135

[18] H. K. Bruce Dunn , J.‐M. Tarascon , Science 2011, 334, 928. 22096188 10.1126/science.1212741

[19] I. Chung , B. Lee , J. He , R. P. Chang , M. G. Kanatzidis , Nature 2012, 485, 486. 22622574 10.1038/nature11067

[20] P. K. Nayak , S. Mahesh , H. J. Snaith , D. Cahen , Nat. Rev. Mater. 2019, 4, 269.

[21] G. Wang , F. S. Melkonyan , A. Facchetti , T. J. Marks , Angew. Chem. Int. Ed. Engl. 2019, 58, 4129. 30395372 10.1002/anie.201808976

[22] T. R. Ray , J. Choi , A. J. Bandodkar , S. Krishnan , P. Gutruf , L. Tian , R. Ghaffari , J. A. Rogers , Chem. Rev. 2019, 119, 5461. 30689360 10.1021/acs.chemrev.8b00573

[23] C. E. Zhao , P. Gai , R. Song , Y. Chen , J. Zhang , J. J. Zhu , Chem. Soc. Rev. 2017, 46, 1545. 28211932 10.1039/c6cs00044d

[24] Q. Shi , B. Dong , T. He , Z. Sun , J. Zhu , Z. Zhang , C. Lee , InfoMat 2020, 2, 1131.

[25] J. Sun , K. Tu , S. Büchele , S. M. Koch , Y. Ding , S. N. Ramakrishna , S. Stucki , H. Guo , C. Wu , T. Keplinger , J. Pérez‐Ramírez , I. Burgert , G. Panzarasa , Matter 2021, 4, 3049.

[26] L. Gu , L. German , T. Li , J. Li , Y. Shao , Y. Long , J. Wang , X. Wang , ACS Appl. Mater. Interfaces 2021, 13, 5133. 33471495 10.1021/acsami.0c20703

[27] Q. Shi , Z. Zhang , Y. Yang , X. Shan , B. Salam , C. Lee , ACS Nano 2021, 15, 18312. 34723468 10.1021/acsnano.1c07579

[28] Z. L. Wang , A. C. Wang , Mater. Today 2019, 30, 34.

[29] C. Wu , A. C. Wang , W. Ding , H. Guo , Z. L. Wang , Adv. Energy Mater. 2018, 9, 1802906.

[30] P. Yang , Y. Shi , S. Li , X. Tao , Z. Liu , X. Wang , Z. L. Wang , X. Chen , ACS Nano 2022, 16, 4654. 35171554 10.1021/acsnano.1c11321

[31] Q. Zhang , T. Jin , J. Cai , L. Xu , T. He , T. Wang , Y. Tian , L. Li , Y. Peng , C. Lee , Adv. Sci. 2022, 9, e2103694. 10.1002/advs.202103694 PMC8811828 34796695

[32] T. Guo , G. Liu , Y. Pang , B. Wu , F. Xi , J. Zhao , T. Bu , X. Fu , X. Li , C. Zhang , Z. L. Wang , Extreme Mech. Lett. 2018, 18, 1.

[33] M. Chen , X. Li , L. Lin , W. Du , X. Han , J. Zhu , C. Pan , Z. L. Wang , Adv. Funct. Mater. 2014, 24, 5059.

[34] X. Cheng , Y. Song , M. Han , B. Meng , Z. Su , L. Miao , H. Zhang , Sens. Actuator A Phys. 2016, 247, 206.

[35] C. Chen , L. Chen , Z. Wu , H. Guo , W. Yu , Z. Du , Z. L. Wang , Mater. Today 2020, 32, 84.

[36] J. Li , Z. Xie , Z. Wang , Z. Lin , C. Lu , Z. Zhao , Y. Jin , J. Yin , S. Mu , C. Zhang , W. Gui , X. Liang , J. Wang , W. Ding , Nano Energy 2023, 112, 108473.

[37] C. He , W. Zhu , B. Chen , L. Xu , T. Jiang , C. B. Han , G. Q. Gu , D. Li , Z. L. Wang , ACS Appl. Mater. Interfaces 2017, 9, 26126. 28707896 10.1021/acsami.7b08526

[38] L. Ma , R. Wu , S. Liu , A. Patil , H. Gong , J. Yi , F. Sheng , Y. Zhang , J. Wang , J. Wang , W. Guo , Z. L. Wang , Adv. Mater. 2020, 32, 2003897. 10.1002/adma.202003897 32803825

[39] A. Yu , W. Wang , Z. Li , X. Liu , Y. Zhang , J. Zhai , Adv. Mater. Technol. 2020, 5, 1900978.

[40] Y. Yang , Q. Shi , Z. Zhang , X. Shan , B. Salam , C. Lee , InfoMat 2022, 5, e12360.

[41] Y. C. Lai , J. Deng , S. L. Zhang , S. Niu , H. Guo , Z. L. Wang , Adv. Funct. Mater. 2016, 27, 1604462.

[42] Z. Lin , Q. He , Y. Xiao , T. Zhu , J. Yang , C. Sun , Z. Zhou , H. Zhang , Z. Shen , J. Yang , Z. L. Wang , Adv. Mater. Technol. 2018, 3, 1800144.

[43] I.‐W. Tcho , W.‐G. Kim , S.‐B. Jeon , S.‐J. Park , B. J. Lee , H.‐K. Bae , D. Kim , Y.‐K. Choi , Nano Energy 2017, 42, 34.

[44] G. Zhao , Y. Zhang , N. Shi , Z. Liu , X. Zhang , M. Wu , C. Pan , H. Liu , L. Li , Z. L. Wang , Nano Energy 2019, 59, 302.

[45] Q. Shi , Z. Zhang , T. He , Z. Sun , B. Wang , Y. Feng , X. Shan , B. Salam , C. Lee , Nat. Commun. 2020, 11, 4609. 32929087 10.1038/s41467-020-18471-z PMC7490371

