# Supplementary material for: Multimodal Intelligent Flooring System for Advanced Smart‐Building Monitoring and Interactions
Source: Adv Sci (Weinh). 2024 Aug 22;11(40):2406190. doi: 10.1002/advs.202406190 (PMC11516046; doi:10.1002/advs.202406190)
Supplement: Supplementary file 1 — Supporting Information [file ADVS-11-2406190-s004.docx]

Supporting Information

Multimodal Intelligent Flooring System for Advanced Smart-Building Monitoring and Interactions

Yuqi Chen, Jianlong Hong, Yukun Xiao, Huiyun Zhang, Jun Wu*, Qiongfeng Shi*

Y. Chen, J. Hong, Y. Xiao, H. Zhang, Prof. J. Wu, Prof. Q. Shi

Joint International Research Laboratory of Information Display and Visualization, School of Electronic Science and Engineering, Southeast University, Nanjing 210096, China

E-mail: wujunseu@seu.edu.cn, qiongfeng@seu.edu.cn

**Table S1.** Comparison table of the flooring systems based on TENG.

| **Ref** | **Sensor**  **type** | **Sensing**  **signal** | **Connection method** | **Number of array units** | **Number of output channels** | **Neural network** | **Limitation** |
| --- | --- | --- | --- | --- | --- | --- | --- |
| **1** | Planar | Position | Connected separately | 24 | 24 | No | Complex array realization; Unimodal |
| **2** | 3D structure | Position | Row and column connections | 4 | 4 | No | Complex array realization; Unimodal |
| **3** | Planar | Position & Pressure | Connected separately | 8 | 8 | No | Complex array realization; Planar sensors are not suitable for pressure detection |
| **4** | Planar | Position | Connected in parallel for group with varying electrode cover rates | 12 | 2 | Yes | Unable to recognize inclined walking; Unimodal |
| **5** | Planar | Position | Connected in parallel for group with coding | 16 | 8 | Yes | To recognize inclined walking, the number of output channels should be increased to 16; Unimodal |
| **6** | Planar & 3D structure | Position & Pressure | Connected in parallel for group with varying IDE widths | 24 | 8 | Yes | Position information and pressure information are acquired separately |
| **This work** | Planar & 3D structure | Position & Pressure & Material (Multimodal information are acquired at the same time) | Connected in serial for group with resistors | 16 (can extend to 64 and beyond) | 8 (remains constant as the array is scaled up) | Yes | Accuracy of the system needs to be improved; Energy consumption of the system needs to be reduced |


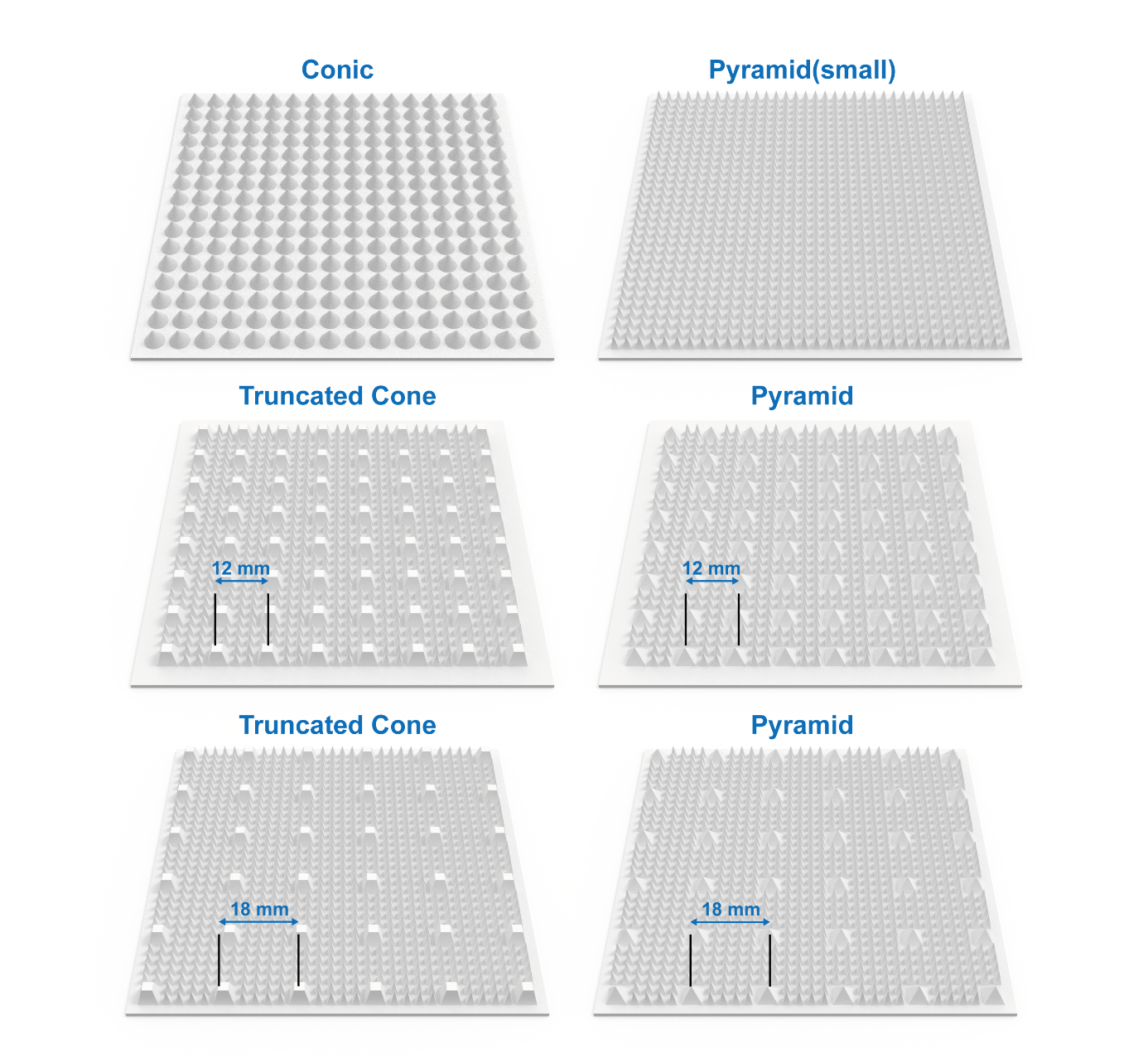


**Figure S1.** The detailed schematic diagram of six different microstructures.


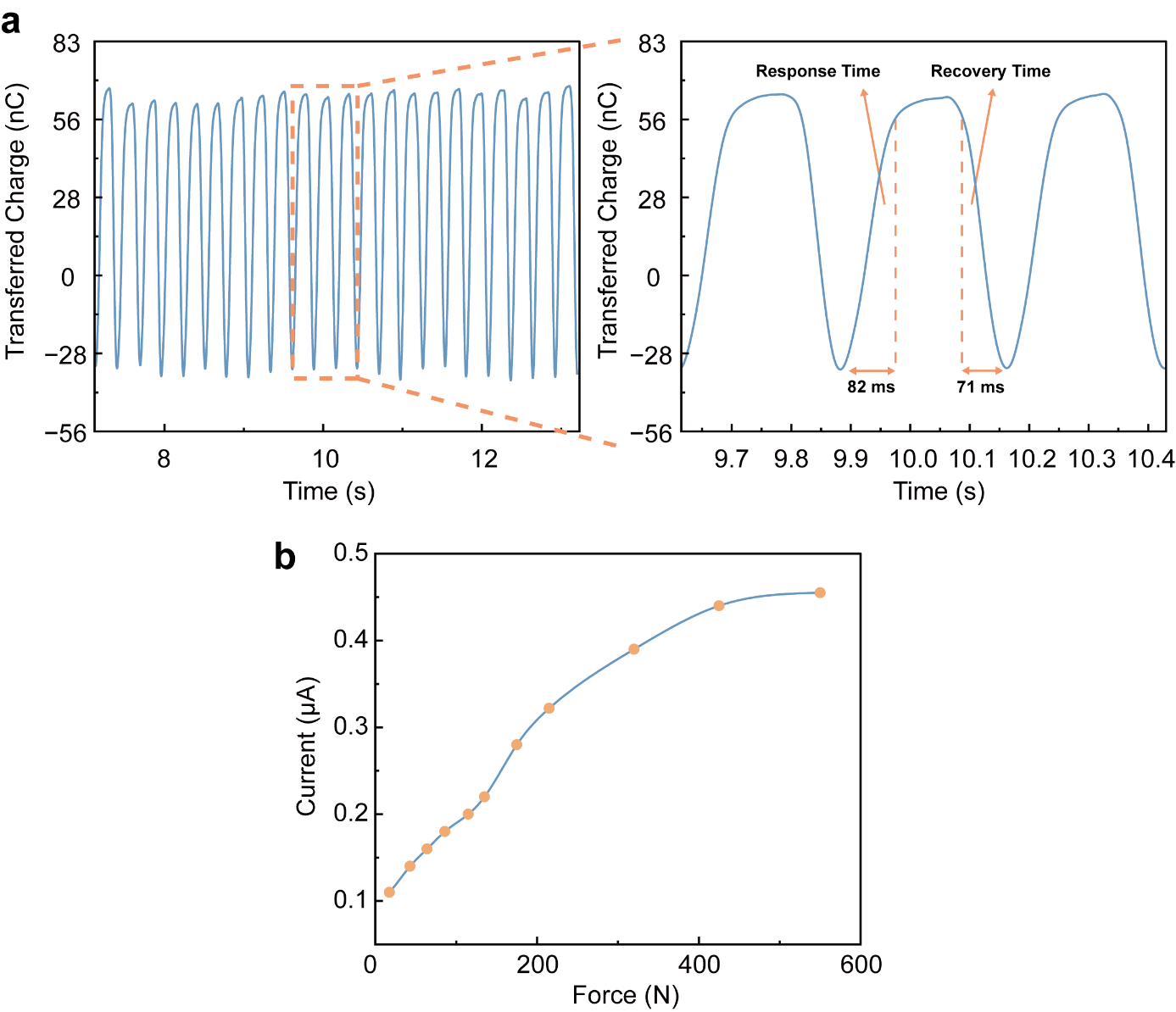


**Figure S2.** Other characteristics of the ultra-elastic triboelectric pressure sensor. (a) The response time and recovery time of the pressure sensor. (b) The current of the pressure sensor.


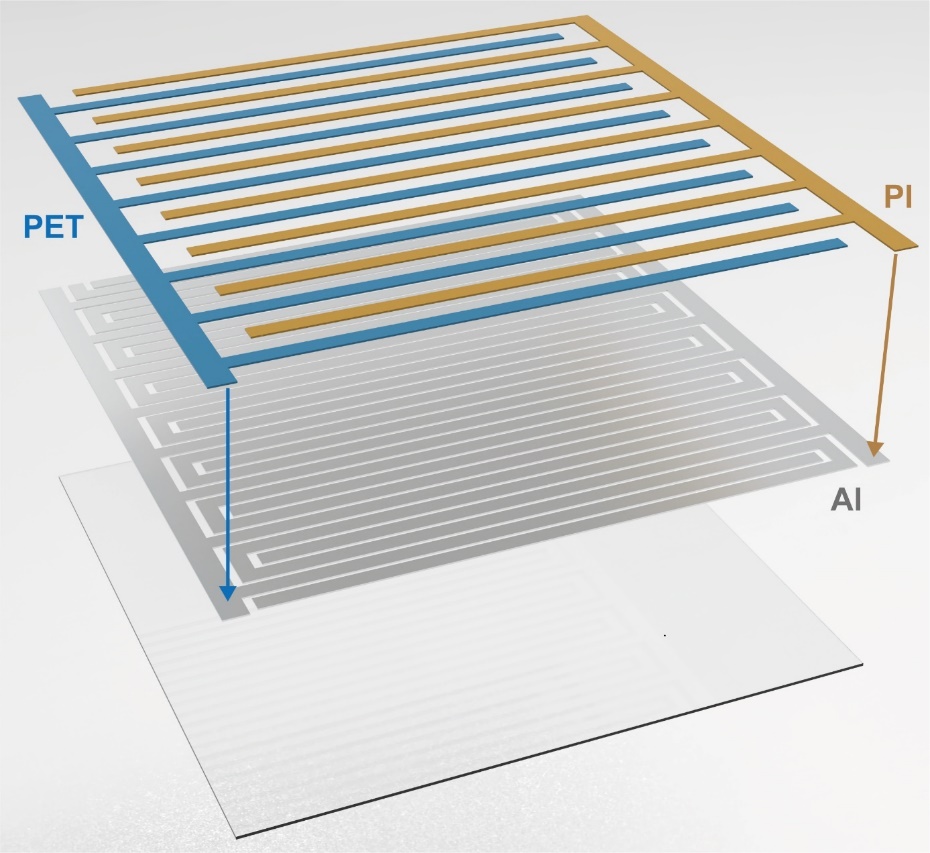


**Figure S3.** Electrode composition of the material sensor.


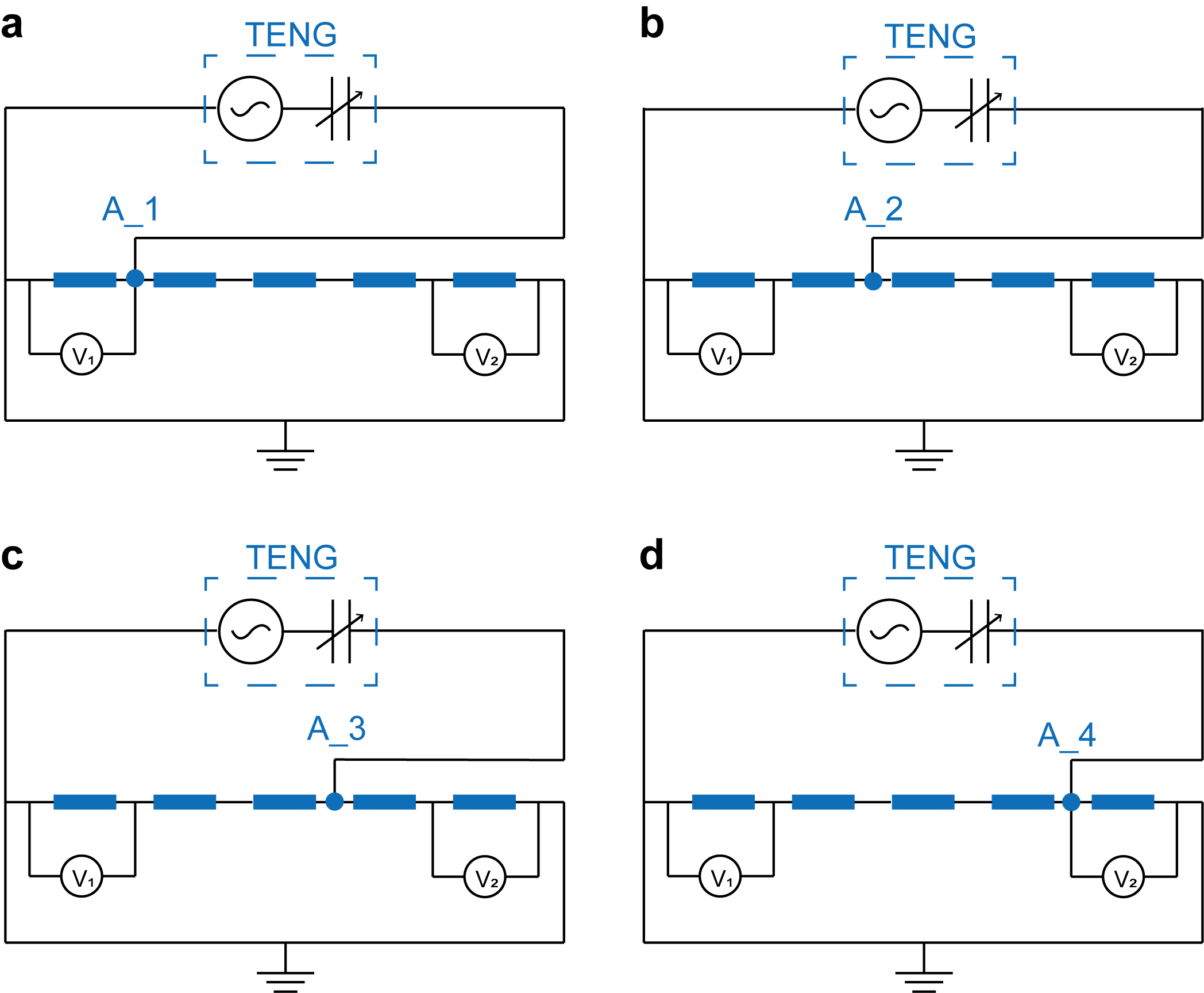


**Figure S4.** Localization equivalent circuit for Group A. (a) Stepping on A_1. (b) Stepping on A_2. (c) Stepping on A_3. (d) Stepping on A_4.


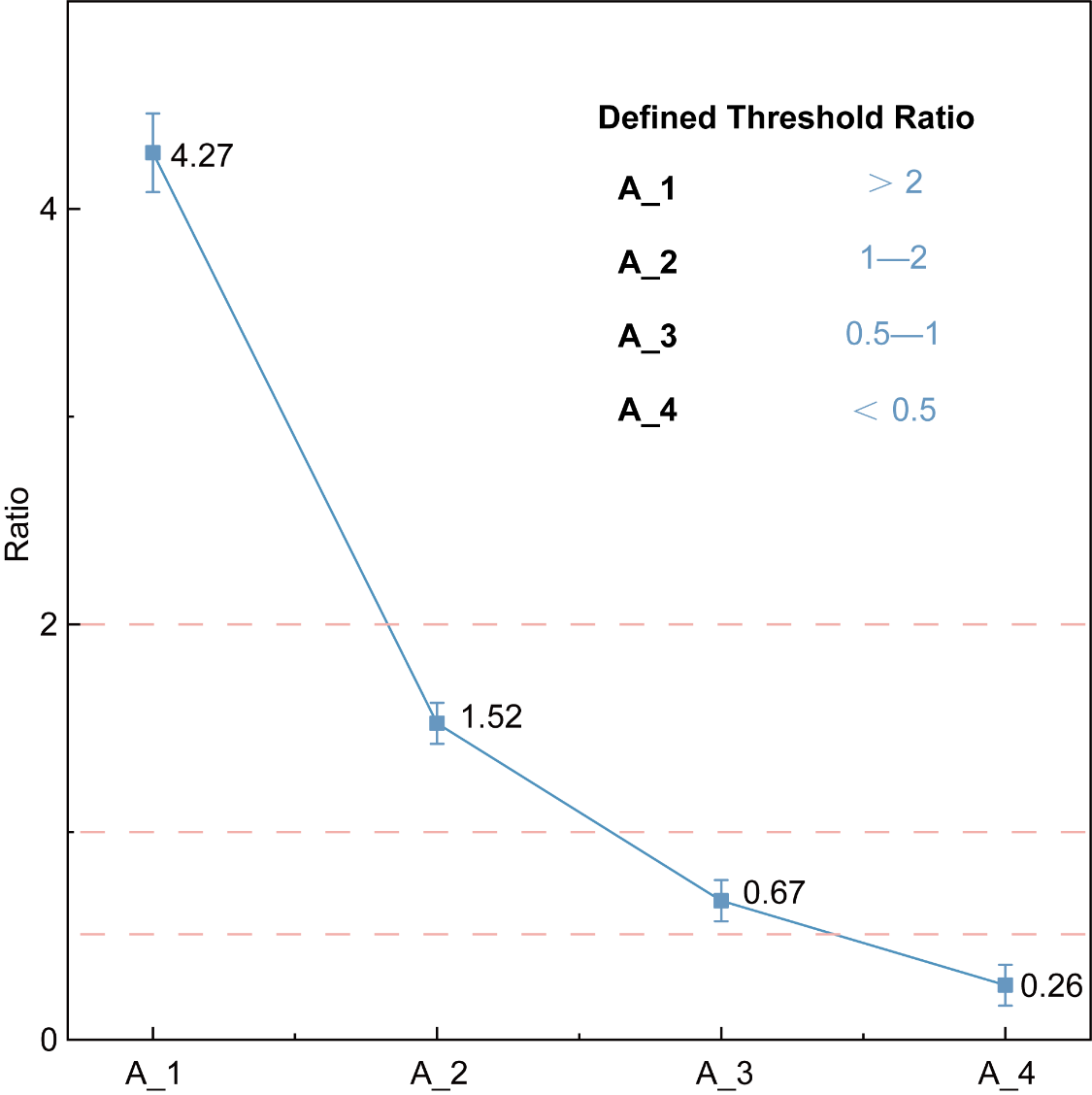


**Figure S5.** Average values and standard deviations of the voltage ratios when stepping on the four positions in Group A.

**
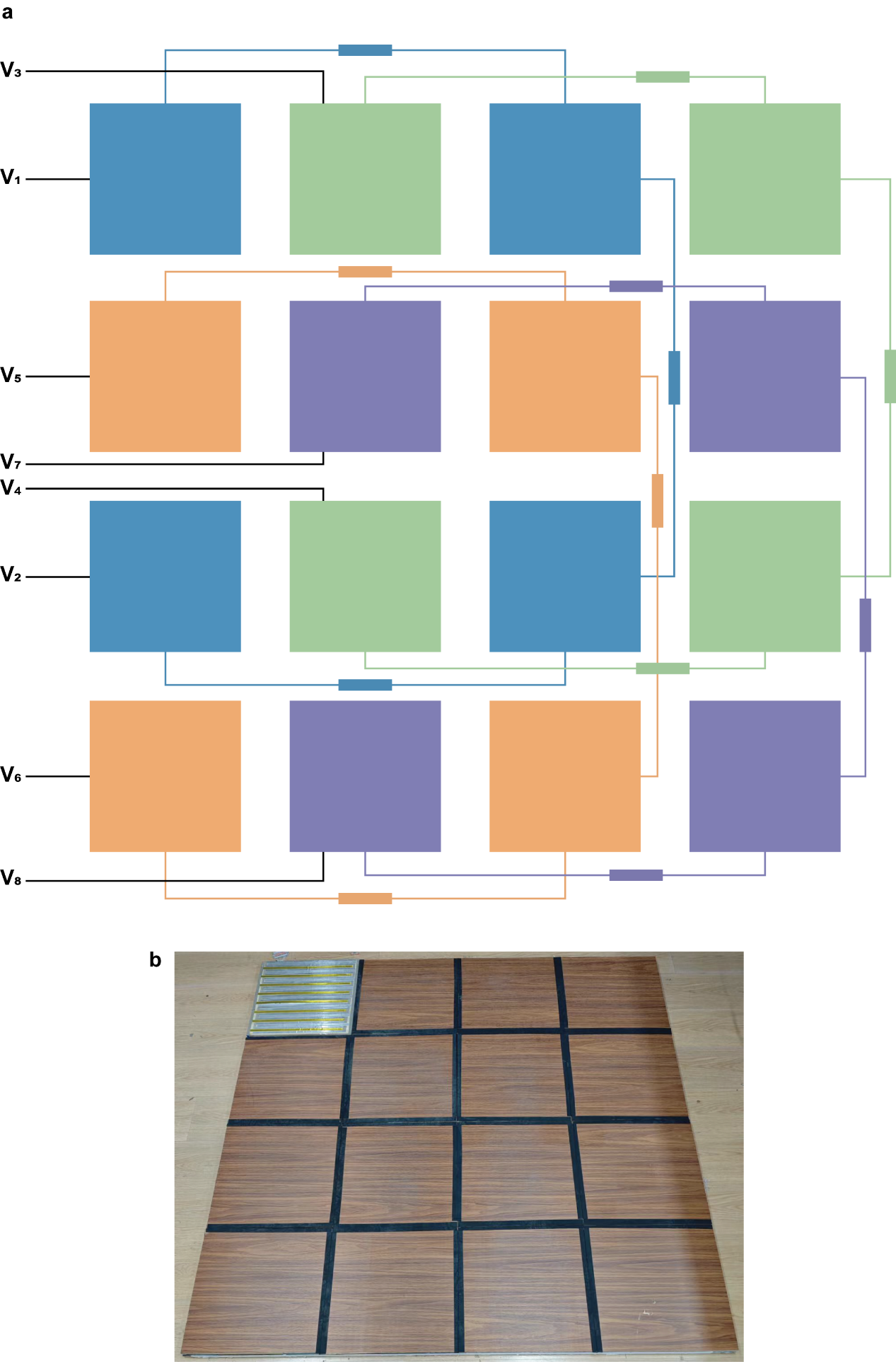
**

**Figure S6.** The illustration of the connected floor array. (a) The schematic illustration of the electrode connection and unit arrangement. (b) The photograph of the proposed floor array.


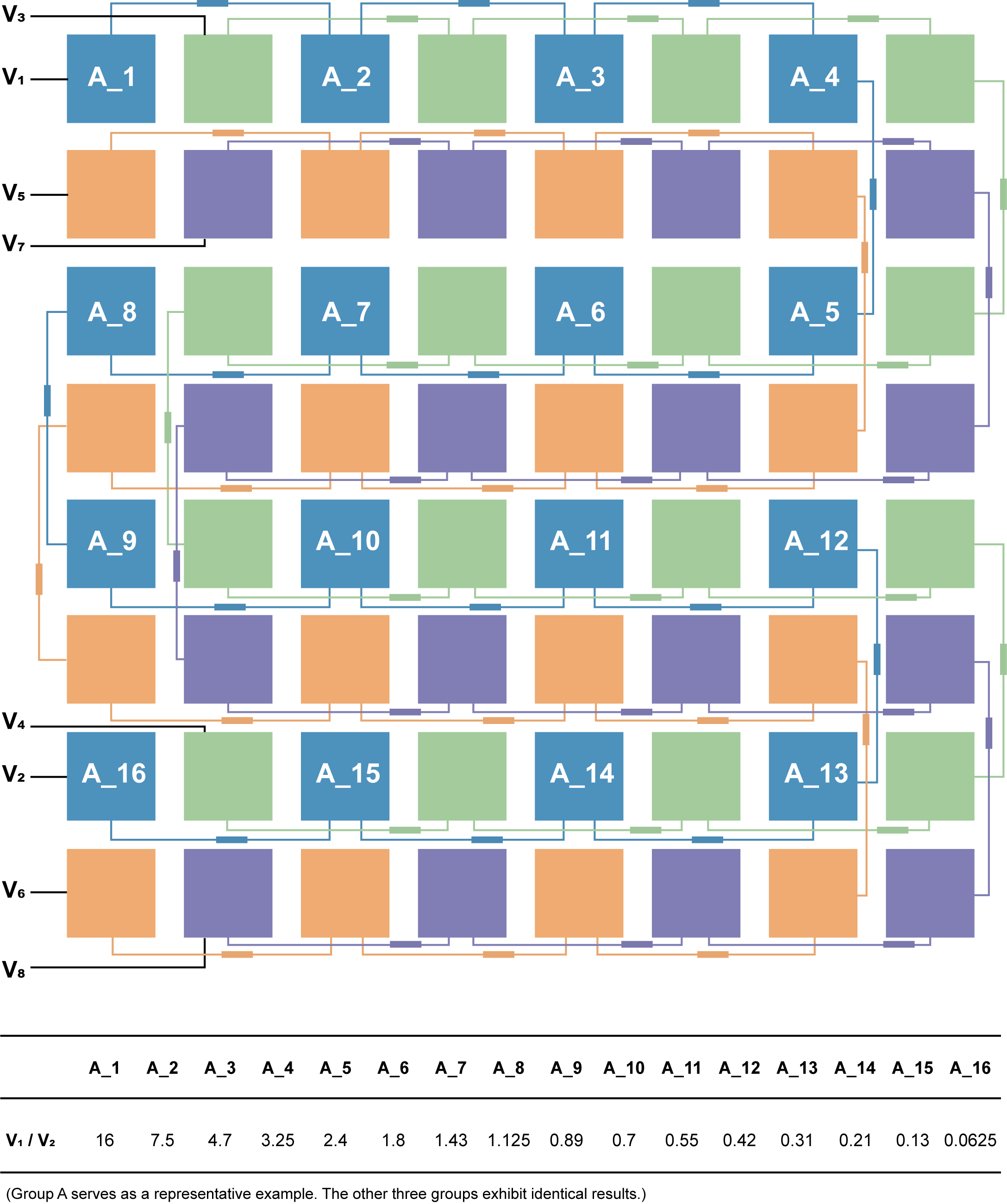


**Figure S7.** The schematic illustration of the electrode connection and unit arrangement for an 8×8 floor array.


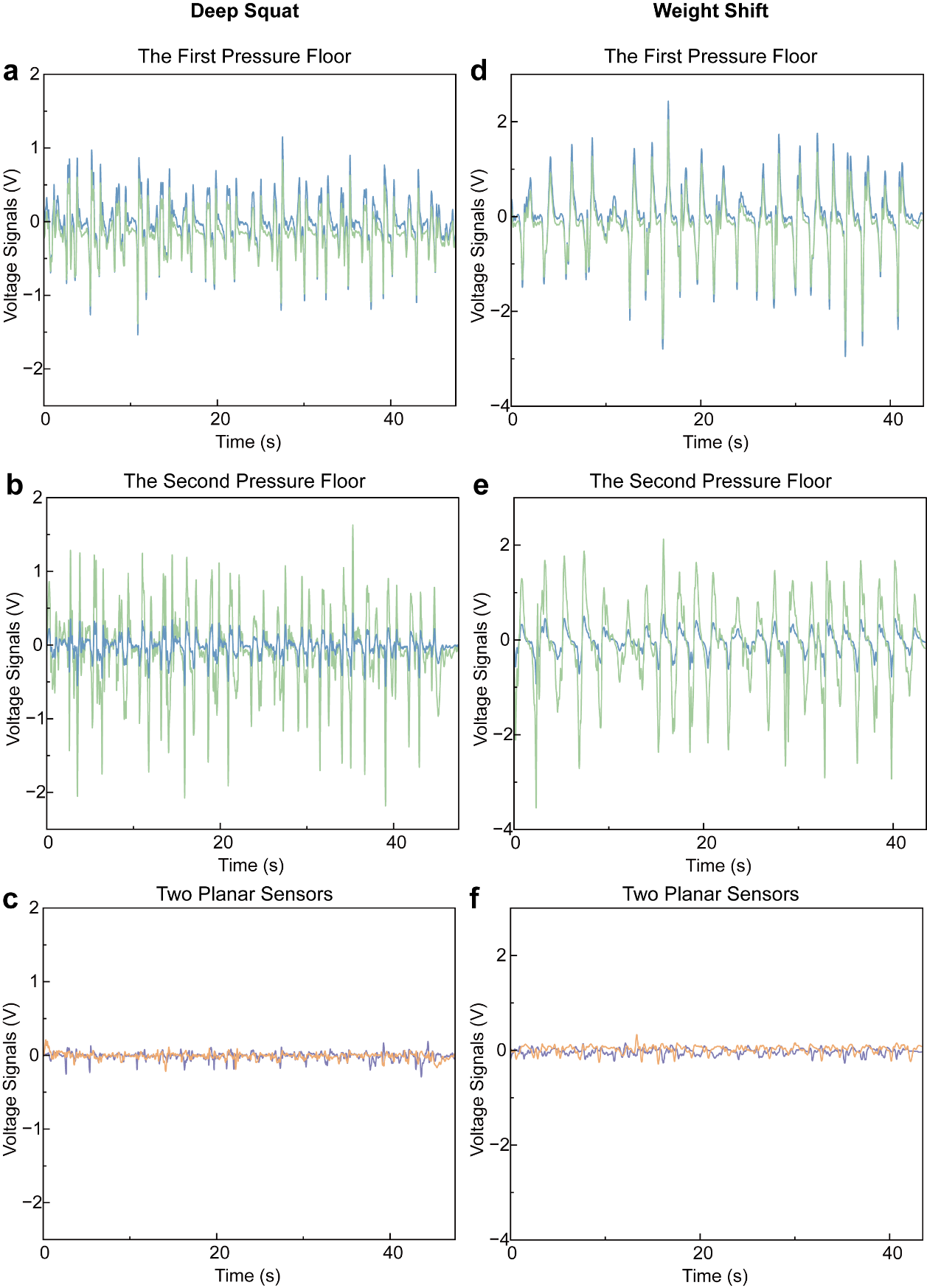


**Figure S8.** The voltage signals from deep squatting and weight shifting. (a) Voltage signals from the first pressure floor when deep squatting. (b) Voltage signals from the second pressure floor when deep squatting. (c) Voltage signals from the two planar sensors when deep squatting. (d)-(f) Voltage signals when weight shifting.

**
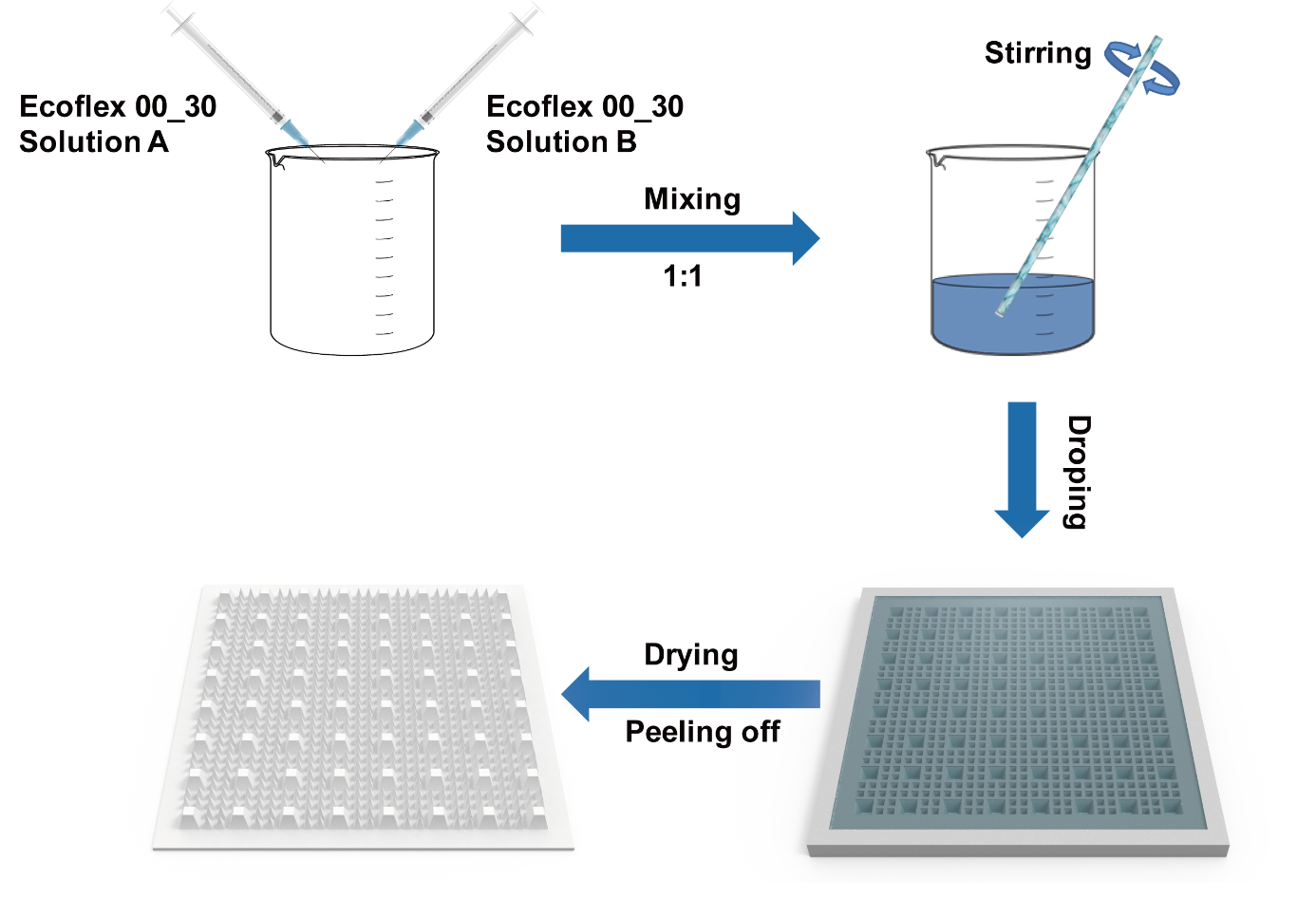
**

**Figure S9.** The microstructure formation process of the negative triboelectric layer.

References

[1] A. Yu, W. Wang, Z. Li, X. Liu, Y. Zhang, J. Zhai, Adv. Mater. Technol. 2020, 5 (2), 1900978.

[2] X. Cheng, Y. Song, M. Han, B. Meng, Z. Su, L. Miao, H. Zhang, Sens. Actuator A Phys. 2016, 247, 206.

[3] J. Ma, Y. Jie, J. Bian, T. Li, X. Cao, N. Wang, Nano Energy 2017, 39, 192.

[4] Q. Shi, Z. Zhang, T. He, Z. Sun, B. Wang, Y. Feng, X. Shan, B. Salam, C. Lee, Nat. Commun. 2020, 11, 4609.

[5] Q. Shi, Z. Zhang, Y. Yang, X. Shan, B. Salam, C. Lee, ACS Nano 2021, 15 (11), 18312.

[6] Y. Yang, Q. Shi, Z. Zhang, X. Shan, B. Salam, C. Lee, InfoMat 2022, 5 (1), e12360.
